# Remote sensing and geospatial approach: Optimizing groundwater exploration in semi-arid region, Nepal

**DOI:** 10.1016/j.heliyon.2024.e31281

**Published:** 2024-05-17

**Authors:** Sandesh Dhakal, Rajan Subedi, Saroj Kandel, Saurav Shrestha

**Affiliations:** a Tribhuvan University, Institute of Forestry, Pokhara Campus, Pokhara, Nepal; b University of New Haven, 300 Boston Post Road, West Haven, CT, 06516, USA

**Keywords:** Bayesian weight of evidence, GIS, Modified Dubois and Topps model, Mustang, Soil moisture

## Abstract

Groundwater is the fundamental component of the hydrological system that acts as a major factor in comprehensions of the physical processes in both the land surface and the atmosphere. Determining groundwater, which directly affects the agricultural productivity of semi-arid mountainous regions, is crucial. Mountain ecosystems, once abundant with flowing water, now face immense pressure from a changing climate, evident in the drying of springs and the diminishing flow of groundwater. Ensuring a steady flow of water, fair access to it, and responsible use are the cornerstones of a secure future for mountain communities. This study aims to assess the groundwater potential zones using remote sensing and a geospatial approach in the Mustang Valley's rural municipalities (Thasang and Gharapjhong). Nine factors were assigned to assess the groundwater potential map: slope, drainage density, lineament density, geology, soil, land use/land cover, rainfall, aspect, and soil moisture. The Bayesian weights of evidence model was used to delineate the groundwater potential zone. The study categorized groundwater potential across the region, creating five zones: very high, high, moderate, low, and very low. These zones covered 0.6 %, 12.4 %, 51.2 %, 35.5 %, and 0.3 % of the study area. The accuracy of the groundwater potential map was assessed by comparing its predictions with the actual locations of springs, using the area under the curve metric. The receiver operating characteristics curve analysis yielded an area under the curve of 0.7226, indicating a 72.26 % accuracy in predicting the presence of groundwater. The findings of this paper contribute to a better understanding of groundwater potential zones, which can support policymakers and planners for hydrological, meteorological, and crop planning applications in this climatically vulnerable region.

## 1 Introduction

Groundwater is the water found in aquifers, which can store and transmit groundwater, providing a reliable source during drought [1]. Groundwater acts as a vital component for both ecological well-being and human adaptability, playing a crucial role in a fluctuating environment [2]. Soil moisture (SM) and groundwater are the crucial terrestrial variables that govern the energy and mass balance in interactions between the land and the atmosphere [3]. They are also essential to human existence, social and economic growth, and the protection of natural systems [4]. Currently, due to global climate change and other anthropogenic factors, soil moisture content and water resources are under stress [5]. Agriculture productivity is highly dependent on SM, and a shortage of SM can cause a sizable loss in crop yield [6]. This crucial problem of SM measurement has been considered less of a key research area. Similarly, a United Nations (UN) report published in 2023 has forewarned people about the impending water problem and its potentially harmful consequences [7]. In such cases, groundwater has become a crucial resource for supplying different sectors with the water they need including domestic, irrigation, and industries [8]. Therefore, for groundwater resources to be developed in a balanced and sustainable manner, a precise quantitative evaluation based on reasonably accurate scientific concepts was necessary. Furthermore, remote sensing and geospatial technologies enable the acquisition and application of substantial volumes of granular data across extensive areas, with adjustable spatial and temporal resolutions [9].

A vital part of the hydrological system, groundwater is found in subterranean geological formations called aquifers. It ranks among its most valuable and accessible natural resources, supporting biological diversity, economic growth, and human health [10]. The results of the 2021 national census reveal that more than 57 % of Nepalis rely on agriculture, forestry, or fishing for their livelihoods [11]. Nepal is one of the climatically vulnerable countries that has faced climate-induced problems such as an increase in temperature and uncertainty of rainfall [12]. Climate change casts an undeniable shadow over the drinking water sector, posing significant challenges [13]. The presence and attainability of groundwater were impacted by the replenishment mechanism, which was regulated by a range of factors encompassing physiographic attributes, lithological composition, arrangement of drainage systems, land utilization, land covering, alongside climatic elements like temperature, rainfall, evapotranspiration, and geological characteristics such as fractures and lineament attributes [14]. The arid and semi-arid regions face intensifying water scarcity due to inadequate surface water availability [15]. In numerous mountainous regions, observations have indicated a decline in the activity of springs and seepage that primarily rely on groundwater sources, leading to their drying up in recent times. Moreover, several villages have been forced to relocate from different parts of study regions due to the scarcity of water [16]. From the perspective of climate change, such a situation was predicted to get considerably worse in the future. Therefore, it was necessary to search for an alternative water source i.e., groundwater which was the principal source of drinking among 23.3 % of people in the mountain region of Nepal [17]. Therefore, our study focuses on the identification of groundwater potential zones using multi-variate spatial analysis.

For an effective implementation of irrigated agriculture, water availability determination was crucial, particularly in arid or semi-arid locations where crop development and yield may be negatively impacted by water scarcity and poor water quality. Extensive hydrogeological research and utilization endeavors have taken place in Nepal within the Indo-Gangetic plain, particularly aimed at fulfilling the requirements for irrigation and potable water supply [18]. Nevertheless, hydrogeological exploration within mountainous regions remained relatively understudied, despite the significant reliance of mountain communities on groundwater sources for household purposes. Groundwater constituted an essential element of the subterranean geological structures (aquifers) that formed part of the hydrological system. To assess the potential groundwater reservoirs effectively, researchers adapt and apply various direct and indirect techniques depending on the specific local context [19]. Mapping groundwater potential with high accuracy was most effectively achieved through an indirect approach that leveraged satellite data, remote sensing technologies, and geographic information systems [20]. Remote sensing offers a benefit through its provision of data with spectral, spatial, and temporal attributes, which can be acquired swiftly even in remote and hard-to-reach regions. Consequently, remote sensing has evolved into a highly useful instrument for gaining entry to, safeguarding, and overseeing groundwater reserves [21].

Satellite imagery has long been a proven tool for accurately mapping and extracting diverse land characteristics, including soil moisture, drainage density, geology, slope, soil type, land use/cover, aspect, and lineament [22,23]. Over the year, researchers have explored a diverse range of methodologies, including analytic hierarchy process (AHP), fuzzy logic, multiple criteria decision analysis (MCDA), frequency ratio, BWOE, and machine learning, to identify promising groundwater zones [[24], [25], [26], [27], [28], [29]]. Golla et al. (2022) leveraged twelve weighted factors and ArcGIS 10.1's for overlay analysis to create a groundwater potential map of India's semi-arid Anantapur region [30]. Similarly, using geographic information system (GIS) and weighted overlays, Pathak et al. (2016) analyze five factors (lineament density, geology, drainage density, slope, and rainfall) to identify promising groundwater zones in Nepal's mountainous rocky aquifers [31]. Moreover, Pathak (2017) and Pathak et al. (2019) delineate the groundwater potential (GWP) zone in an Indo-Gangetic plain and the western Daraudi river basin in Nepal through GIS analysis [32,33]. The comparative analysis by Maity et al. (2022) for GWP zone shows that Bayesian weight of evidence (BWOE) model performed more accurately than the AHP model and was also less biased [28]. Similarly, Bayesian weight of evidence (BWOE) was the most relevant method to calculate the groundwater potential based on the varying significance of influencing factors [28]. According to BWOE, standardized contrast τ is regarded as a confidence measure, where a higher τ value indicates more GWP and vice versa. Furthermore, the BWOE model proved to be an appropriate method for assessing the reliability of the outcome, thereby diminishing partiality in the decision-making procedure [34]. The integration of these layers can yield a rapid and advantageous delineation of zones with potential for groundwater resources [35]. This study employs a BWOE model, integrating nine diverse factors (slope, soil type, lineament density, drainage density, rainfall, land use and land cover, aspect, geology, and soil moisture), to map the groundwater potential of the study area. So, this paper accesses the groundwater potential zone using multi-variate spatial analysis. Finally, we used spring source data to validate the obtained map using the area under the ROC curve (AUC). Therefore, this paper can support efficient settlement planning and natural resource management in this region in the future.

## 2 Materials and methods

### 2.1 Study area

**Fig. 1:**
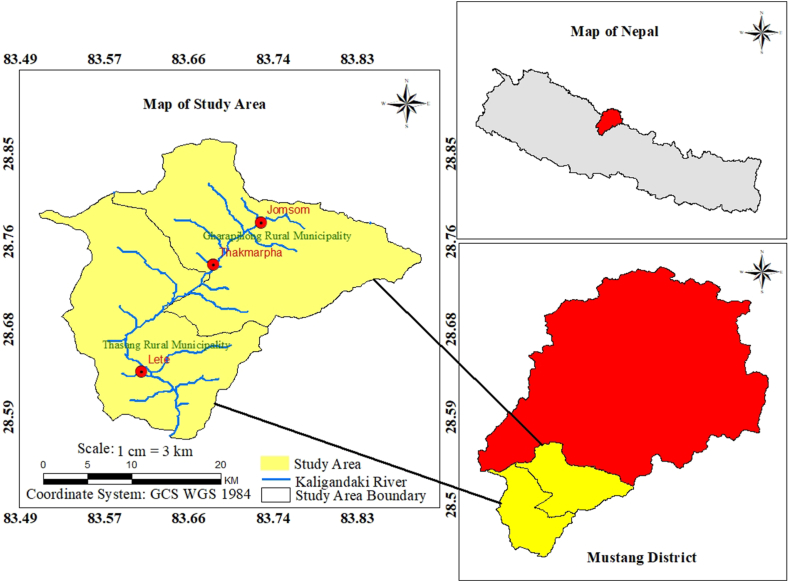
Map of the study region.

The study was carried out in Thasang and Gharapjhong, two rural municipalities in Mustang district, Gandaki province, western Nepal. The area covers 577.97 square kilometers and has a population of 6568 [11]. It is located between 28°33' - 28° 52′ Northern latitudes and 83° 28′ 30'' - 83° 53′ Eastern longitudes. The elevation range varies from 1,372 m to 2863 m, with features such as elevated mountains, river valleys, spurs, saddles, vividly colored stratified rock formations, barren high-altitude semi-arid, ridges, and valleys. The Kali Gandaki River cuts through the heart of the study area, originating from the central region (Fig. 1). This area represents the essence of the Trans-Himalayan zone with its unique combination of a continental climate and substantial temperature fluctuations. It is also vulnerable to climate change [36], and its impact, such as the precipitation of increased snowfall together with rapid melting triggers more frequent flash floods [37]. The region is sheltered from the effects of the monsoon since it is on the side that receives less rain. This region receives an average annual rainfall of 1097 mm, concentrated primarily in the summer monsoon season with minimal precipitation during the winter. This declining precipitation in Lete (28° 37′ 57.83" – 83° 36′ 33.19″, 2490 m alt.), annual precipitation is around 1813 mm, decrease to below 531 mm in Thakmarpha (28° 44′ 27.27″– 83° 40′ 53.81″, 2695 m alt.), also drops to 300 mm in Jomosom (28° 47′ 2.43″–83° 43′ 47.34″, 2740 m alt.). The majority of snowfall occurs in the winter and is over 2500 m in elevation, however, the area can experience intense snowfall at any time throughout the year. As a result of the Lee and Foehn effects, the northern part of the study area receives progressively less rain throughout the year. Water, then, is always the resource that triggers human survival in these parched uplands. Water is a fundamental and scarce resource in arid upland areas, playing a pivotal role in the survival, livelihoods, and well-being of the local population. The primary source of income for the rural people in the study region is agriculture, which is mostly dependent on it for food security. Consequently, the security of local livelihoods depends on agricultural output, the availability of water in the area, access to water resources, and their sustainable management. Similarly, there is an increase in demand for water from new settlements (lodges), expanding fruit agriculture and tourism. To prevent future water shortage issues and economic loss in this region, a deeper knowledge of freshwater resources, particularly groundwater, is important. This study establishes a critical foundation of data for decision-makers, allowing them to delve into the complexities and craft sustainable solutions for managing groundwater resources in these two rural municipalities.

### 2.2 Data collection

This study relies on remotely sensed images, the data from related governmental organizations, International non-governmental organizations (INGOs), and the United States Geographical Survey (USGS) which were used for groundwater prospect zone mapping are mentioned below in detail.

#### 2.2.1 Digital elevation models (DEM)

According to Dewitt et al. (2015), the USGS shuttle radar topographic mission (SRTM) DEM is an excellent resource for extracting topographic variables [38]. Thus, leveraging a high-resolution digital elevation model (DEM) with 30-m pixels was employed to create several theme maps representing topographic features, including slope, aspects, drainage, and lineament density.

#### 2.2.2 Land use land cover (LULC)

The LULC data used in the study were directly sourced from the latest release (April 2022) of the land cover map produced by the International Centre for Integrated Mountain Development [39].

#### 2.2.3 Geological map

The geological information employed in this analysis was obtained from the USGS World Geological map, available online at https://certmapper.cr.usgs.gov/data/apps/world-maps.

#### 2.2.4 Soil type map

To characterize the soil types, the research utilized data acquired from the International soil reference and information center (ISRIC) soil data hub, a comprehensive online resource accessible at https://www.isric.org/explore/isric-soil-data-hub.

2.2.5. Rainfall: The department of hydrology and meteorology in Gandaki province, Pokhara, provided precipitation data from January 1991 to December 2022, which formed the basis for constructing the average rainfall map of the study area. The study region consists of three meteorological stations in Lete, Marpha, and Jomsom at elevations of 2490 m, 2655 m, and 2741 m. These precipitation data were obtained every month. The monthly data were re-analyzed and converted into average precipitation on a yearly basis. Then these average annual precipitation data were used as input in ArcGIS 10.8 to retrieve the average rainfall map of the study area.

#### 2.2.5 Soil moisture

The data from both Sentinel-1 and Sentinel-2 satellites, were processed through the Google Earth Engine platform (GEE), to estimate soil moisture using the modified Dubois model and Tops model. This study has used 709 S1 images and made the average composite of an interferometric wide (IW) mode image from January 2015 to December 2022, which was in VV polarization with an incidence angle ϴ (value range from 38° to 45°) to derive the average soil moisture index map of the study area. Similarly, in addition to being chosen for the average composite, 940 Sentinel-2 photos were further processed in order to calculate the normalized difference vegetation index (NDVI) utilizing the red (R) and near-infrared (NIR) bands. Table 1 shows the characteristics of sentinel 1 and sentinel 2 images used for soil moisture estimation. The VV polarization band of sentinel 1 GRD data and Red and NIR of sentinel 2 MSI data from January 2015 to December 2022 all seasons were taken for the average soil moisture index map of the study area.

**Table 1:** Characteristics of multi-temporal sentinel data used in soil moisture study.

Region of Interest	Image series	Year of date	Season	Band	Total number of bands used in mega file data cubes
Thasang and Gharapjhong rural municipality Mustang district	Sentinel-1 GRD Sentinel-2	January 2015 to December 2022 (Composite)	All-season	VV Red and NIR	1 2

### 2.3 Methodology

**Fig. 2:**
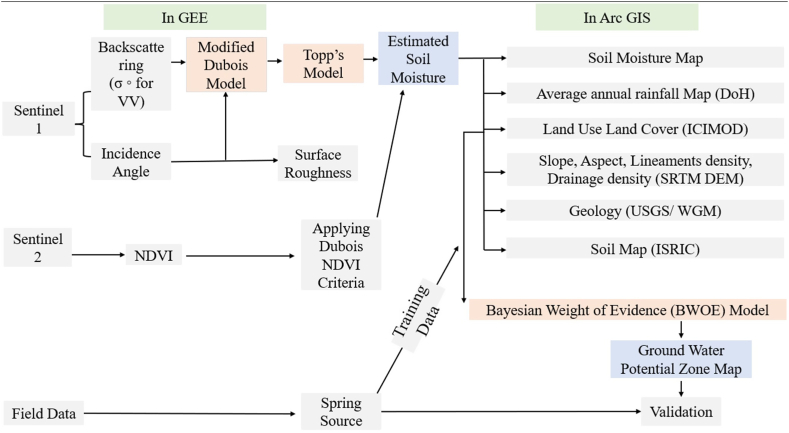
The flow chart depicts the methodology used to identify Groundwater Potential zone.

Here, to evaluate the groundwater potential zone of the study area, nine thematic factors such as, rainfall map, geological map, LULC map, lineaments density map, soil map, drainage density map, slope map, aspect map and soil moisture map, have been used and the data was analyzed in Arc GIS 10.8 software. The overall methodology for groundwater potential zone identification was performed in both Google Earth Engine and ArcGIS 10.8 as shown in Fig. 2. The average soil moisture map was estimated in the GEE platform using modified Dubois and Topp's Model and the retrieved soil moisture index map was further combined with other eight factors for groundwater potential study in ArcGIS (Fig. 2).

#### 2.3.1 Rainfall map

Among various climatic factors, rainfall played a crucial role in identifying areas with high groundwater potential. The study utilized data from the department of hydrology and meteorology to generate an average annual precipitation map, which was then categorized into seven distinct groups based on rainfall variations (ranging from 380.4 mm to 1814 mm). There is less average yearly precipitation in the northern regions of the research area due to their higher altitudes and mountainous zones (elevation of around 2741 m). While lower in elevation (2490 m), these regions of the study area seem to have a noticed an increase in average annual precipitation, with the amount remaining relatively consistent across these areas.

#### 2.3.2 Geological map

Geology plays a major role in shaping groundwater movement and storage, both above and below ground, making it crucial to consider in any groundwater investigation [40]. The geological composition of the region comprises seven distinct classes: Carboniferous and Cretaceous sedimentary rocks, Quaternary sediments, Tertiary igneous rocks, and metamorphic and sedimentary formations of Triassic, Jurassic, and undifferentiated Paleozoic origin. Rocks like Carboniferous sedimentary rocks, Quaternary sediments, and Jurassic metamorphic and sedimentary rocks allow water to seep in more easily than others. This makes them prime targets for identifying areas with high groundwater potential.

#### 2.3.3 LULC map

Land use and cover significantly impact groundwater, including its location, flow pattern, infiltration rate, and its quality. The LULC map was adopted from a secondary source (ICIMOD, 2022). The Google Earth Engine platform was employed using machine learning algorithms and data from Landsat satellites to identify and classify the various land uses within the study area. The initially detailed landcover map with 11 categories underwent simplification, resulting in a 9-class version encompassing distinct landscapes like water bodies, glaciers, snow, forests, built-up areas, croplands, bare soil, grasslands, and other woodlands. The major portion of the study area falls into grassland (254.88 km^2^), snow (95.37 km^2^), and bare soil (83.81 km^2^). The area of other classes includes forest (64.15 km^2^), glacier (26.01 km^2^), other woodland (23.70 km^2^), cropland (16.76 km^2^), water (7.51 km^2^) and built-up area (5.78 km^2^). Priority is assigned to water bodies, forests, and agricultural land, as they significantly contribute to groundwater replenishment through increased infiltration. Conversely, minimal importance is attributed to bare rocks or soil due to their limited capacity for infiltration.

#### 2.3.4 Soil map

The ability of water to infiltrate the ground and contribute to groundwater recharge depends heavily on the specific characteristics of the soil, such as its texture, composition, and structure [41]. The primary considerations for estimating the rate of inflation are the hydraulic properties and soil texture. Consequently, a key determining element in the delineation of the GWP zone was the properties of the soil. The soils of the region can be classified into four major categories: Gelic Leptosols, characterized by thin layers and cold temperatures; Rock outcrops, areas with exposed bedrock; Eutric Regosols, young soils with minimal development; and Humic Cambisols, soils with a darker surface layer enriched in organic matter. Based on their rate of infiltration, soil has been ranked. Eutric Regosols and Humic Cambisols have higher infiltration potential than Gelic Leptosols and areas with rock outcrops [42]. Hence, soil with a higher infiltration capacity rate is given higher priority, while soil with the lowest infiltration rate is assigned low priority.

#### 2.3.5 Lineaments density map

The density of lineaments is measured as the total length of all lineaments within a specific area [43]. Here, the SRTM DEM was used to extract the lineament using the hill-shades effect. The image processing techniques, i.e., generating multiple hill-shade images from DEM by varying azimuth and altitude angles were performed, which created different lighting conditions to highlight various aspects of the terrain. In this work, the lineament density was extracted using these lineaments.

(1) Ld=∑i=1i=nLiA

where, equation (1): reveals the relationship between the total length of lineaments (Li) within a region, the area (A) of that region, and the resulting lineament density (Ld). This study employed four classes to categorize lineament density, ranging from 0 to 0.37, 0.38–0.75, 0.76–1.10, and 1.20–1.50 fractures per kilometer squared. The “high density” zone signifies an abundance of fractures within a unit area, while the “low density” zone indicates a significantly lower concentration. High lineament density promotes groundwater recharging and is therefore preferable to low lineament density in terms of groundwater potential.

#### 2.3.6 Drainage density map

The drainage density of a basin is found by dividing the total length of its streams by the basin's area. More streams (higher drainage density) mean more water can infiltrate into the ground, leading to better groundwater recharge [43]. Using ArcGIS software, the drainage density map was taken from the SRTM Digital Elevation Model (DEM). The study divided areas into four drainage density zones: low (0–0.52), medium-low (0.53–1), medium-high (1.1–1.6), and high (1.7–2.1). They found that areas with more streams (higher density) tend to have better groundwater recharge potential (GWP), and vice versa.

#### 2.3.7 Slope map

The topography of Thasang and Gharapjhong rural municipalities features rolling landscapes that transition from flat plains to steep hillsides. Slope characteristics across the region were derived from a high-resolution (30 m × 30 m) digital elevation model. The study region was stratified into four discrete slope classes using a digital elevation model. The most dominant class (class 2), representing 32.97 % of the total area, encompasses slopes with angles between 150 and 300°. The infiltration potential increases as the slope angle decreases. The flatter areas have a higher capacity to absorb water than steeper slopes.

#### 2.3.8 Aspect map

Slope orientation, known as aspect, is a quantitative measure of the compass direction it faces, represented by degrees ranging from 0° (north) to 360° with 90° representing east, 180° south, and 270° west. The land in this area primarily slopes downward in the northern, northeastern, and eastern directions. Areas with slopes facing south and east tend to get more rain, making them more likely to have groundwater beneath them.

#### 2.3.9 Soil moisture map

The soil moisture map was derived using Sentinel-1 and sentinel-2 images using the modified Dubois model and Tops model (Fig. 2). The backscattering coefficient of single VV polarization of sentinel-1image was used and run to the modified Dubois model to determine the relative soil permittivity which was further used in Topps model to retrieve the volumetric surface soil moisture in m^3^/m^3^. The limitation of the Dubois model was that it couldn't correctly classify soil moisture over the dense forest with an NDVI greater than 0.4. Therefore, Sentinel-2 images were used for the NDVI map, and the Soil moisture index value over NDVI >0.4 was masked. A similar method by Parida et al. (2022) was followed in this study for soil moisture calculation [44]. The soil moisture data underwent a reclassification process, resulting in five distinct classes. Four of these classes are defined by specific ranges of moisture content values, while the fifth class encompasses areas with missing or unreliable data that remain unclassified. The assumption was, that the reason for having a high average soil moisture value has a higher potential for GW availability.

### 2.4 Bayesian weight of evidence (BWOE) model

Within the realm of groundwater potential assessment, the Bayesian weights of evidence (BWOE) model ranks as a frequently employed methodology [[45], [46]]. The BWOE model leverages the relative significance of various conditioning factors to estimate the spatial distribution of groundwater potential (GWP) across the study area, resulting in a GWP map.

(2) $$W^{+}=\mathrm{LN}\frac{P\left(\frac{B}{A}\right)}{P\left(\frac{B}{A^{\prime }}\right)}$$

(3) $$W^{-}=\mathrm{LN}\frac{P\left(\frac{B^{\prime }}{A}\right)}{P\left(\frac{B^{\prime }}{A^{\prime }}\right)}$$

where P is the probability, B is the conditioning factor, B′ is the absence of conditioning factors, A is the spring source data, and A′ is the absence of spring source data. W+ and W− signify the positive and negative weights of the conditioning factors which were computed using equation (2) and equation (3).

Next, the contrast value's standard deviation (SD) may be computed using equation (4):

(4) $$SD_{C}=\sqrt{\left(\left(S^{2}\;\left(W^{+}\right)+S^{2}\;\left(W^{-}\right)\right)\right)}$$

where, where S^2^ represents the conditioning elements' affecting weight [47]. Standardized contrast τ, which is regarded as a confidence metric, may be computed in the manner described by equation (5):

(5) $$\tau =\left(\frac{C}{SDc}\right)$$

where C is weight contrast. Once all conditioning parameters have been assigned using the spring source data, the values of W+, W−, SDc, and τ have been computed.

The groundwater potential index (GPI) was calculated according to the τ value using equation (6):

(6) GPI=τ1+τ2+……….+τn…

where τ is the weight that is ultimately determined by computing W+, W−, C, and SDc. Then, the GPZ was calculated according to this index value as follows (equation (7)):

(7) GWPZ=∑(Gτ+SLτ+Sτ+DDτ+LULCτ+Rτ+LDτ+Aτ+Smτ)

where G stands for geology, SL for slope, S for soil distribution, DD for drainage density, LULC for land use and cover of the research area, R for rainfall, LD for lineament density, A for aspect, and Sm for soil moisture.

### 2.5 Validation

As previously stated, the spatial distribution of spring sources served as ground truth data for validating the GWP map generated using the BWOE model. A ROC curve analysis was performed to evaluate the model's performance in predicting spring locations, and the AUC value was calculated to summarize this performance [48]. The receiver operating characteristic (ROC) curve depicts the trade-off between the model's ability to correctly identify actual spring locations (true positive rate) and incorrectly classifying non-spring areas as having water (false positive rate). True positive rates increase on the y-axis, while false positive rates increase on the x-axis, both represented as cumulative percentages. The groundwater productivity data set (i.e., categorized by the model) and the true data set (i.e., verification dataset, spring location) were compared to create this curve. The AUC value, calculated from the entire ROC curve, provided a concise measure of the model's overall prediction accuracy. The rate indicates how accurately the factor and model forecast the occurrence [49].

## 3 Results and discussions

**Fig. 3:**
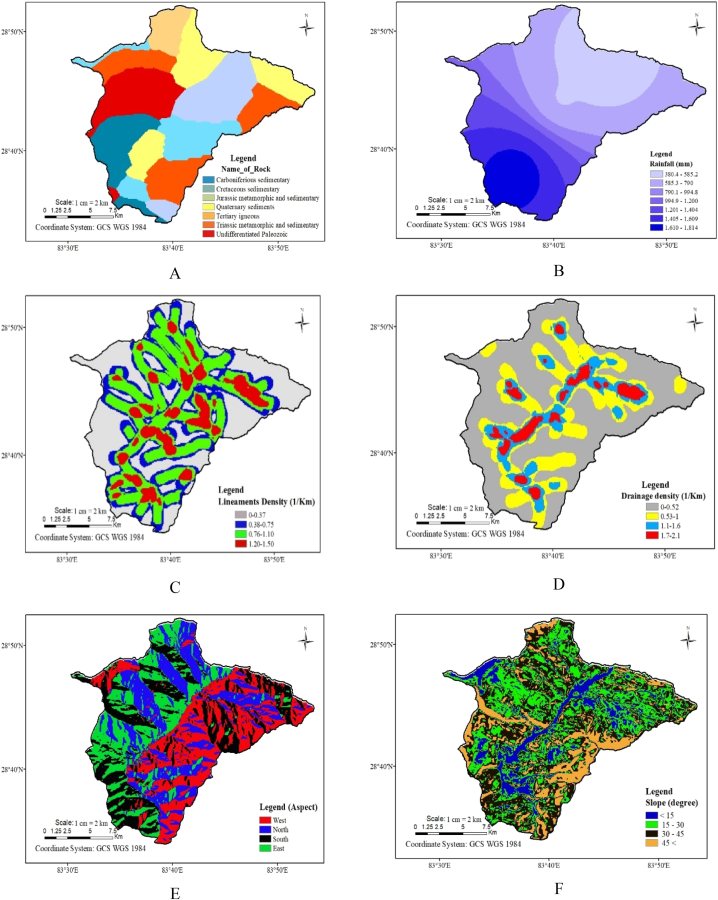

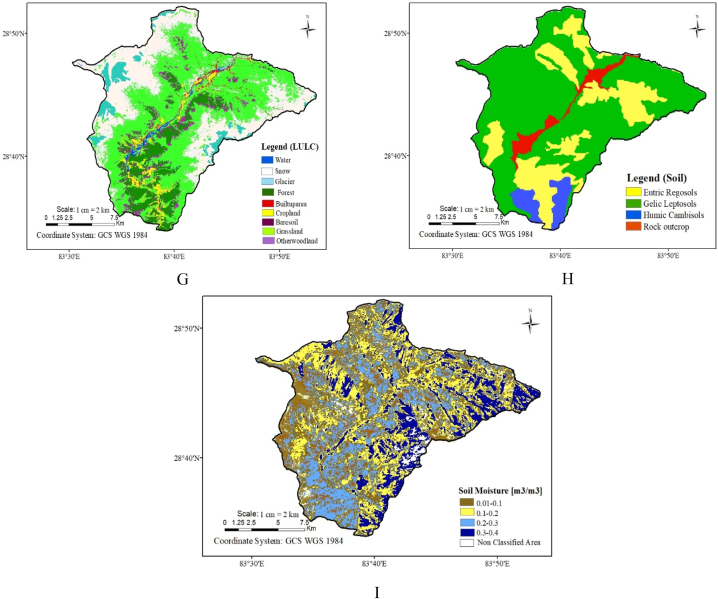
Nine thematic layers for groundwater potential map (A) Geological Map, (B) Rainfall Map, (C) Lineament density Map, (D) Drainage density Map, (E) Aspect Map, (F) Slope Map, (G) LULC Map, (H) Soil Map and (I) Soil moisture map.

Fig. 3 Displays the various data types (thematic layers) we used in this study. Here, figure A is the geological map, B is the rainfall map, C is the lineament density map, D is the drainage density map, E is the aspect map, F is the slope map, G is the land use land cover map, H is the soil map and I is the soil moisture map. Moreover, rankings were designated to these thematic layers, along with assigning weights to individual classes within each layer. After converting all data layers (thematic maps) into a raster format, the data was combined to produce a single output representing the entire study region. This combined output was then classified into different categories to create a map showing the potential area for groundwater.

### 3.1 Relative weight of various thematic layers and classes

The BWOE approach was employed to deduce and compute spatial correlation metrics like W+, W-, and C, which linked the groundwater productivity dataset (spring sources) with each influencing variable. The pertinent variables for each scenario were chosen for groundwater productivity potential mapping based on their spatial correlation values (refer to Table 2). Binary patterns representing predictors for each variable were allocated specific weights, which were then integrated using the overlay functions within ArcGIS as per equation (7). Subsequently, these integrated patterns were combined across various factors and endowed with respective weights. Additionally, as indicated by the BWOE model (τ) values in Tables 2 and it is worth noting that the factors with greater influence on groundwater potential were assigned the highest τ values, signifying heightened groundwater potential. The presence of spring in each particular factures defines the value of τ which then further classifies the identical region representing other GWP. It was defined based on the presence and absence of springs which were used as the training sets. The area having the higher possibility of groundwater has received a high value of τ whereas the area having a low groundwater possibility receives a low value of τ as shown in Table 2.

**Table 2:** The spatial relationship between spring locations and τ BWOE model.

Thematic Layers	Attribute details	Number of pixels in a class	% of pixels in a class	Number of pixels of spring sources	% of pixel of spring source	W+	W-	C (w++w-)	SDc	τ(C/SDc)
Slope (Degree)	<15	166264	25.89	7	58.33	2.032	−0.681	2.713	0.394	6.891
	15–30	211731	32.97	3	25	3.121	−1.089	4.21	0.589	7.148
	30–45	138071	21.5	1	8.33	3.792	−0.568	4.36	0.199	−0.03
	>45	126127	19.64	1	8.33	3.363	−0.327	3.69	0.206	1.47
Soil	Eutric Regosols	132163	20.58	6	50	1.975	−0.484	2.441	0.254	−0.52
	Gelic Leptosols	353591	55.06	4	33.33	2.176	−0.389	2.564	0.204	3.56
	Humic Cambisols	77384	12.05	2	16.67	3.691	−1.558	5.249	0.272	−4.28
	Rock outcrop	79054	12.31	0	0	3.235	−0.28	3.514	0.307	−0.31
Lineaments Density (1 /Km)	0–0.37	223483	34.8	2	16.67	2.592	−0.287	2.879	0.26	−1.76
	0.38–0.75	122273	19.04	2	16.67	3.38	−0.276	3.579	0.227	−0.2
	0.76–1.10	214107	33.34	3	25	0.61	−0.126	0.736	0.099	−1.13
	1.20–1.50	82201	12.8	5	41.66	0.828	−0.07	0.898	0.245	2.92
Drainage Density (1 /Km)	0–0.52	371500	57.85	1	8.33	3.911	−0.672	4.583	0.42	−5.68
	0.53–1	158740	24.72	2	16.67	3.004	−0.49	3.493	1.005	−0.57
	1.1–1.6	71411	11.12	5	41.67	2.182	−0.686	2.868	0.099	−0.15
	1.7–2.1	40540	6.31	4	33.33	1.887	−0.188	2.075	1.005	−1.82
Rainfall (mm)	380.4–585.2	142577	22.20	2	16.67	3.787	−2.053	5.84	0.222	0.63
	585.3–790	194374	30.27	0	0	2.457	0.361	2.818	0.561	0.642
	790.1–994.8	63169	9.84	0	0	3.231	0.104	3.335	0.523	0.197
	994.9–1200	61191	9.53	0	0	3.114	0.100	3.214	0.525	0.190
	1201–1404	44810	6.98	3	25	1.363	−0.246	1.609	0.411	3.915
	1405–1609	64310	10.01	3	25	1.002	−0.213	1.213	0.402	3.017
	1610–1814	71761	11.17	4	33.33	1.180	−0.333	1.512	0.237	6.380
LULC	Water	8348	1.3	3	25	3.222	−1.336	4.558	0.201	2.020
	Glacier	28899	4.5	1	8.33	2.536	−0.124	2.660	1.005	−2.850
	Snow	105962	16.5	0	0	2.430	−0.109	2.539	0.222	0.550
	Forest	71283	11.1	4	33.33	2.380	−0.518	2.898	0.249	−1.190
	Builtuparea	6422	1.0	1	8.33	3.363	−0.327	3.690	0.206	1.470
	Cropland	18624	2.9	2	16.67	0.551	−0.015	0.566	0.254	0.040
	Baresoil	93118	14.5	0	0	2.343	−0.099	2.441	0.099	−0.020
	Grassland	283207	44.1	0	0	2.721	−0.153	2.874	0.207	−1.960
	Otherwoodland	26330	4.1	1	8.33	3.363	−0.327	3.690	0.206	1.470
Aspect	South	164924	25.68	2	16.67	2.965	−0.464	3.429	0.237	0.990
	West	144310	22.47	4	33.33	2.176	−0.389	2.564	0.204	3.560
	North	165078	25.71	3	25	2.578	−0.466	3.044	0.222	0.630
	East	167881	26.14	3	25	1.887	−0.188	2.075	1.005	−1.820
Geology	Undifferentiated Paleozoic rocks	92820	14.45	1	8.33	3.792	−0.568	4.360	0.199	−0.030
	Tertiary igneous rocks	36659	5.71	1	8.33	3.363	−0.372	3.690	0.206	1.470
	Quaternary sediments	116962	18.21	4	33.33	2.176	−0.389	2.564	0.204	3.560
	Cretaceous sedimentary rocks	85455	13.31	1	8.33	3.282	−0.296	3.578	0.227	−0.200
	Triassic metamorphic and sedimentary rocks	124426	19.38	0	0	2.454	−0.112	2.566	0.349	−0.310
	Jurassic metamorphic and sedimentary rocks	108078	16.83	3	25	1.887	−0.188	2.075	1.005	−1.820
	Carboniferious sedimentary rocks	77793	12.11	2	16.67	2.592	−0.287	2.879	0.260	−1.760
Soil moisture	0.01–0.1	181355	28.24	2	16.67	2.881	−0.416	3.297	0.718	4.594
	0.1–0.2	207556	32.32	3	25	2.716	−0.566	3.282	0.589	5.571
	0.2–0.3	112705	17.55	5	41.67	2.385	−0.72	3.105	0.461	6.736
	0.3–0.4	85219	13.27	2	16.67	3.026	−0.505	3.531	0.718	4.92

### 3.2 Groundwater potential zones

**Fig. 4:**
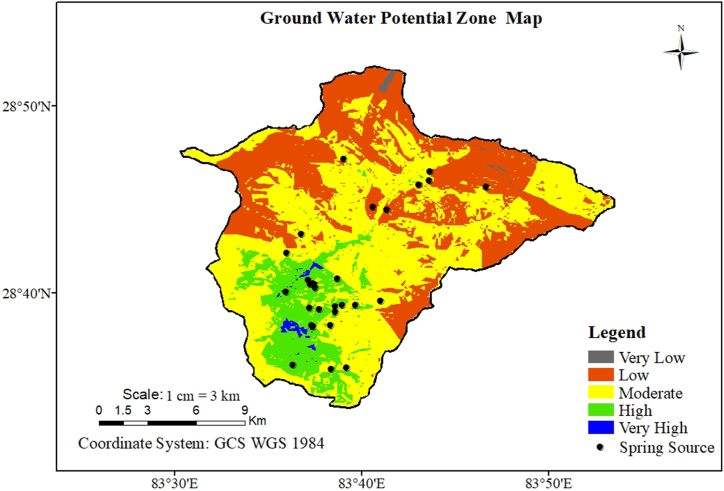
Groundwater potential zone of the study area.

The integration of these nine thematic maps through the BWOE model facilitated the delineation of groundwater potential zones within the study region. The generated map was recategorized into five zones (very low, low, moderate, high, and very high) representing groundwater potential. The threshold for classifying the area with very low to very high GWP was based on the respective GWP index value, as shown in Table 3 and the final map was visualized in Fig. 4.

**Table 3:** Classified Groundwater potential zones.

S. N	Value (GWP Index)	Class
1	11.2–16.53	Very Low
2	16.53–21.25	Low
3	21.25–27.62	Moderate
4	27.62–33.27	High
5	33.27–42.51	Very High

The moderate potential zone (PZ) accounted for the highest area coverage by moderate PZ 51.2 %, followed by low PZ (35.5 %), high PZ (12.4 %), very high PZ (0.6 %), and very low PZ (0.3 %) as shown in Table 4. The spatial distribution of groundwater potential (GWP) depicted by the map revealed that mountainous terrain, snow cover, glaciers, barren areas, and grasslands were categorized as having very low or low GWP values. In contrast, regions in proximity to rivers, agricultural land, and forested areas exhibited high or very high GWP values.

**Table 4:** Percentage coverage of GWP area.

Groundwater Potential	Area in Km^2^	Percentage coverage (%)
Very low	1.73	0.3
Low	205.18	35.5
Moderate	295.92	51.2
High	71.67	12.4
Very High	3.47	0.6
**Total**	**577.97**	**100**

### 3.3 Validation

**Fig. 5:**
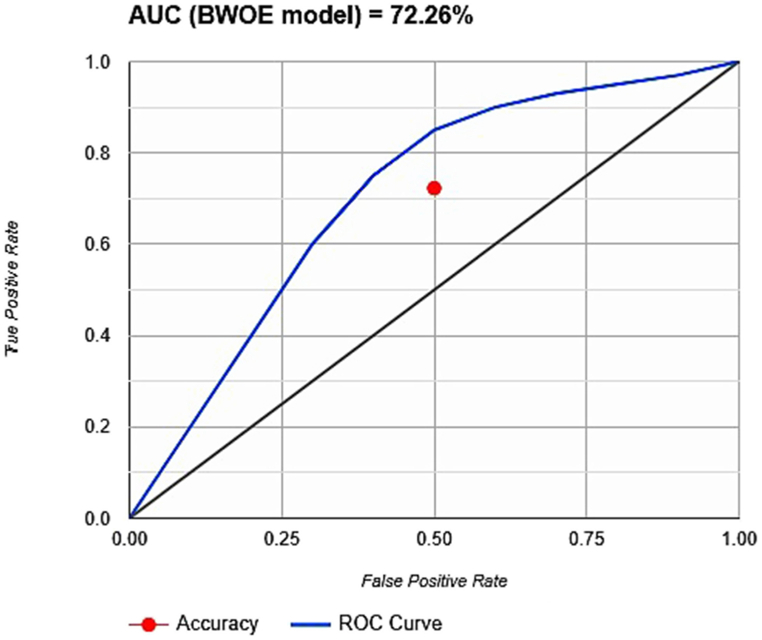
ROC curve for GWPs maps prepared using BWOE model.

Twelve out of twenty-nine groundwater spring source locations that were found were chosen as training samples, and the remaining seventeen spring sources were used for validation, which was in line with the objective. Leveraging the AUC metric derived from the ROC curve enabled a concise evaluation of the projected GWP zone map's accuracy. The AUC value of 0.7226 obtained from the ROC curve analysis suggests a 72.26 % accuracy in predicting groundwater potential zones (Fig. 5). The outcome of the accuracy evaluation indicates that the BWOE model's prediction accuracy was satisfactory in predicting the existence of groundwater in these two rural municipalities (Thasang and Gharapjhong).

### 3.4 Discussions

This research employed remote sensing (RS), geographic information systems (GIS), and a Bayesian Weights of Evidence (BWOE) model to create a map indicating areas with potential groundwater resources. To identify potential areas for groundwater in the Mustang district, two rural municipalities (Thasang and Gharapjhong) were chosen, and an ensemble of Bayesian weights of evidence (BWOE) models was applied. The groundwater potential zone (GPZ) was determined by analyzing nine conditioning factors and input parameters from thematic maps. The GPZ of the models was prepared based on spring source data. The BWOE framework leveraged the spatial distribution of spring locations to calculate weights for each category of the conditioning elements, reflecting their influence on the presence of groundwater. The output maps were classified into five zones, ranging from very low to very high, based on their potential for groundwater resources. According to this methodology, the research area's hilly topography included low potential zones, whereas the center regions and riverine areas had significant groundwater potential.

Remote sensing techniques make it possible for large-scale modeling of groundwater prospect zones. However, limited spatial resolution in remote sensing data, particularly satellite imagery, hinders the accurate identification of small-scale groundwater features like localized aquifers or groundwater recharge zones [50]. The dynamic nature of the hydrological system may not always align with the temporal resolution of the remote sensing data. Similarly, while remote sensing techniques using satellite sensors excel at capturing surface and near-surface features, their limited depth penetration capabilities hinder detailed exploration of deeper groundwater resources [51]. An appropriate model was therefore crucial for the prediction of the GWP with minimum errors. The BWOE model was acknowledged for its ability to estimate groundwater recharge rates; however, it faces limitations stemming from its assumptions of consistent recharge rates and homogeneous soil properties [52], which may not accurately reflect the complexities of varied hydrogeological environments. Despite the constraints mentioned, the combination of field scale measurement together with remote sensing data and methodologies such as the BWOE model remains valuable for understanding groundwater resources.

The distribution of land use and land cover (LULC) and the underlying rock types (lithology) significantly affect the weight of each factor when creating maps of groundwater potential [53]. Previous studies have incorporated eight parameters, LULC, Soil, Geology, Rainfall, Aspect, Slope, Drainage density, and Lineament density for GWP assessment [[25], [26], [27],^33^,^41^]. However, as soil moisture is a critical driver of groundwater availability [54], so we incorporated it as an additional parameter in our study. This study was one of the approaches for the study of groundwater potential zones using spring source location as the training data over the semi-arid region. The findings of this study support similar to Lee et al. (2012), we observed a positive correlation between high lineament density and increased groundwater potential [55]. According to Kumar et al. (2014) and Gizaw et al. (2023), lithological features and lineament density act as pathways and catalysts for water infiltration, contributing to groundwater recharge [56,57]. Similarly, the study by Pathak et al. (2021) found that lineament and hydro-geomorphology are the most influential factors for delineating groundwater potential zones in the mountainous area of the Himalayan region [58].

The accuracy of the GWP map was evaluated using the AUC value calculated from a ROC curve, mirroring the methodology adopted by Rahmati et al. (2016) for groundwater potential validation [59]. This map was a valuable tool for sustainable groundwater management as it identifies areas with high groundwater potential, prioritizes drilling activities, regulates groundwater extraction, and ultimately ensures long-term water security for this study region. GPZ result analysis is useful for locating future land use planning, agricultural development, and preserving locals' resilience to climate change [60]. Nevertheless, the study's findings provide decision-makers with the tools and techniques needed to craft an effective GWP identification based on spring source data and formulation of plan for long-term, sustainable groundwater management.

## 4 Conclusions

This study employed remote sensing and geospatial techniques for both the detection of groundwater potential zones (GWPZs) and the exploration of several promising groundwater prospect regions. This research investigated the combined application of remote sensing, GIS, and the BWOE model for generating a groundwater potential map of the study area. This was achieved through a three-step approach, encompassing the establishment of thematic layers, the determination of weights of factors in the BWOE method, and overlay analysis to define groundwater potential zones. To quantify the influence of various factors on groundwater potential, tau (τ) values were determined for nine key parameters: slope, drainage density, lineament density, soil properties, geology, land cover/land use, rainfall patterns, aspect, and soil moisture content. The final groundwater potential (GWP) map categorized the area into five classes based on potential: very low (0.3 %), low (35.5 %), moderate (51.2 %), high (12.4 %), and very high (0.6 %). The derived GWP map was also valid using the ROC curve. The model was trained using 12 of the 29 identified groundwater spring locations, while the remaining 17 served as the validation data for the final map. The analysis revealed that the GWP map retrieved using the BWOE model classified GWP zones with reasonable accuracy (AUC = 72.26 %). The spatial distribution of the GWP map shows that the region around Jomsom and Syang has seen less groundwater potentiality. The very high groundwater potential was found in the lower ecological belt, which consists of less sloping land and receives high rainfall. Planning professionals rely heavily on GWP maps for selecting appropriate sites for hazard mitigation, water resource management, and exploration projects. Therefore, the government and policymakers can formulate policies for GWP zone mapping which can provide a scientific basis for developing regulations for groundwater extraction and well drilling permits. This can help prevent overexploitation and ensure sustainable groundwater use. Similarly, these techniques may serve as a baseline and guidance for local authorities and planners on their suitable areas for potential groundwater exploration. Additionally, the proposed method for assessing groundwater potential in this hilly terrain of western Nepal might prove beneficial for neighboring areas with comparable geohydrological characteristics.

## Data availability

The data will be made available after the request for the data.

## CRediT authorship contribution statement

**Sandesh Dhakal:** Writing – original draft, Methodology, Investigation, Formal analysis, Data curation, Conceptualization. **Rajan Subedi:** Visualization, Supervision, Conceptualization. **Saroj Kandel:** Validation, Investigation, Data curation. **Saurav Shrestha:** Supervision, Investigation, Formal analysis.

## Declaration of competing interest

The authors declare the following financial interests/personal relationships which may be considered as potential competing interests:Sandesh Dhakal reports financial support was provided by Gandaki Province Academy of Science and Technology. If there are other authors, they declare that they have no known competing financial interests or personal relationships that could have appeared to influence the work reported in this paper.
